# Antero-posterior Duplicate Exstrophy with a Wet Bladder Plate: A Diagnostic Dilemma

**DOI:** 10.21699/jns.v5i3.369

**Published:** 2016-07-03

**Authors:** Nirali Chirag Thakkar, Prince Raj, Yogesh Kumar Sarin

**Affiliations:** Department of Pediatric Surgery, Maulana Azad Medical College, New Delhi

**Keywords:** Exstrophy variant, Bladder Exstrophy Epispadias Complex

## Abstract

Variants of exstrophy are rare anomalies seen in the spectrum of bladder exstrophy-epispadias complex. We present a rare case of duplicate exstrophy with a wet bladder plate. This is a deviation from the classical description of antero-posterior duplicate exstrophy that is associated with a dry bladder plate.

## CASE REPORT

A 1-day-old boy, born of a full term normal vaginal delivery, was referred to us with a defect in lower anterior abdominal wall. The boy had a low lying umbilicus with an exposed bladder plate that was getting soaked with urine, which was being passed in intermittent squirts from a tiny orifice on the bladder plate. He had a well formed phallus and was also passing clear urine per-urethrally in good stream. On insertion of a catheter per-urethrally, the tip of the tube was not seen on the exposed bladder plate. There was no pubic diastasis clinically and on pelvic radiograph (Fig. 1). Hernial orifices, testis and anus were normal. The baby also had polydactyly and thirteen ribs on chest radiograph. Clinically, the differential diagnoses were superior vesical fissure or duplicate exstrophy (antero-posterior). However, the fact that the per-urethral catheter did not come out through the bladder plate went against the diagnosis of superior vesical fissure and a wet bladder plate went against the diagnosis of duplicate exstrophy. A renal ultrasound showed bilateral kidneys normal with a well distended bladder within. Thus a diagnosis of duplicate exstrophy was made. However, there was still a doubt whether one ureter was opening onto the exposed bladder plate. 

**Figure:**
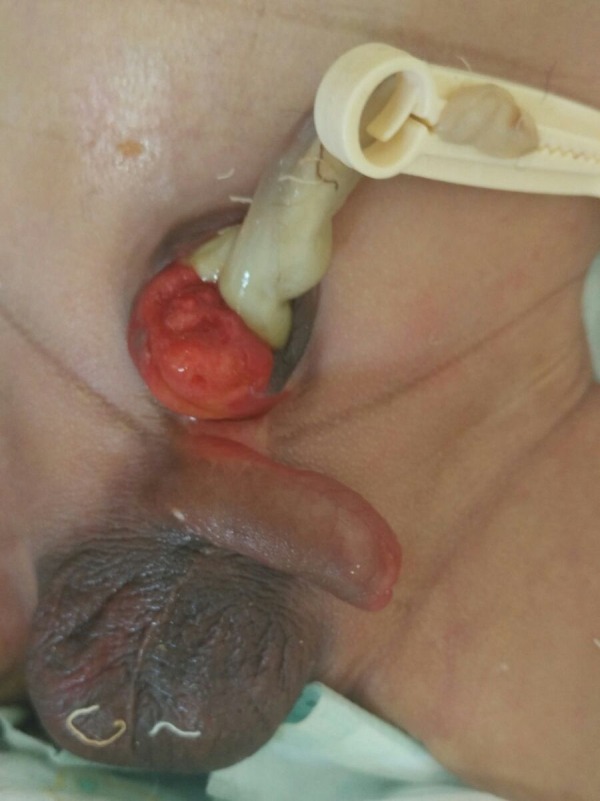
Figure 1: Clinical picture showing bladder mucosa visible on anterior abdominal wall.

The patient was prepared for cystourethroscopy and surgery. On cystoscopy, there was a smooth walled good capacity bladder showing both ureteric orifices ectopically placed, very close to the bladder neck. The left orifice was oval and gaping. On surgical exploration, the visible bladder mucosa on the surface of the abdominal wall was separate from an intact bladder deep to it. The median umbilical ligament was going to the umbilicus which was superior to the duplicate bladder plate. The duplicate bladder plate appeared to have a very tiny communication with the intact native bladder but the communication could not be identified clearly during surgical excision of the bladder plate. Both ureters were draining into the intact bladder. The abdomen could be closed easily without much tension. Per-urethral catheter was removed on post-operative day two and child passed urine in good stream.

## DISCUSSION

Variations of exstrophy are anomalies that do not have all the components of classical bladder exstrophy epispadias complex. They probably have the same embryological origin as classical exstrophy and have some of the defects of classical exstrophy.[1-3] 

Duplicate exstrophy, first described by Marshall and Muecke in 1962, refers to the antero-posterior variety of duplicate exstrophy, where the exstrophied bladder plate is accompanied by a normal phallus and an underlying intact bladder.[4] The ureters do not drain into the exstrophied bladder. A rare case of one ureter draining into the exstrophied bladder plate has been described.[5] There are two similar cases reports in literature, where the clinical presentation was a duplicate exstrophy with a wet bladder plate, creating confusion with a superior vesical fissure.[6,7] The communication in our case was pin-point and was not found on exploration. Its exact etiology is not known.

Exstrophy variants are known to be associated with other congenital malformations like esophageal atresia and tracheo-esophageal fistula, umbilical hernia, anorectal malformations, urethral atresia and limb anomalies.[1] An association with congenital pouch colon has also been reported.[2,3] Our case also had some associated malformations like polydactyly and thirteen ribs, however the relevance of these findings is not known yet.

Variants of exstrophy also need to be followed up for incontinence and may require bladder neck reconstruction.[8] This seems unlikely in our patient as his bladder neck is competent and he is not dribbling. However, he needs to be monitored for vesico-ureteric reflux in view of the cystoscopy findings.

Classical exstrophy and its variants seem to form a continuum. The four variants also are probably not separate entities and there may be significant overlap in clinical presentation.

## Footnotes

**Source of Support:** Nil

**Conflict of Interest:** Nil
